# Southern Chilean Native Plants as Novel Sources of Antioxidant and Antibacterial Extracts

**DOI:** 10.3390/antiox14121488

**Published:** 2025-12-11

**Authors:** Jesús Hernández, Yihajara Fuentes, Eduardo Muñoz-Carvajal, Mario Faúndez, Miguel Gómez, Ady Giordano, Gloria Montenegro

**Affiliations:** 1 Laboratorio de Productos Naturales, Departamento de Ciencias Vegetales, Facultad de Agronomía y Sistemas Naturales, Pontificia Universidad Católica de Chile, Santiago 782-0436, Chile; jhernandez1@uc.cl (J.H.); mgomezu@uc.cl (M.G.); gmonten@uc.cl (G.M.); 2 Departamento de Química Inorgánica, Escuela de Química, Facultad de Química y de Farmacia, Pontificia Universidad Católica de Chile, Santiago 782-0436, Chile; yyfuentes@uc.cl (Y.F.); eamunoz1@uc.cl (E.M.-C.); 3 Departamento de Farmacia, Escuela de Química y Farmacia, Facultad de Química y de Farmacia, Pontificia Universidad Católica de Chile, Santiago 782-0436, Chile; mfaundeza@uc.cl

**Keywords:** antioxidant capacity, antibacterial capacity, reactive oxygen species (ROS), cell viability, bioprospecting, natural extracts

## Abstract

The temperate rainforests of southern Chile host a rich diversity of plants traditionally used in medicine, yet their bioactive potential remains underexplored. This study evaluated the chemical composition, antioxidant capacity, antibacterial activity, and cell viability of ethanolic leaf extracts from *Cissus striata* (CS), *Mitraria coccinea* (MC), and *Raukaua laetevirens* (RL), compared with *Buddleja globosa* (BG), a well-known medicinal shrub. Extracts were obtained using 70% ethanol, ensuring high recovery of polyphenolic compounds while avoiding thermal degradation. The total phenolic content (TPC) was highest in CS, exceeding values reported for green tea, while MC exhibited the greatest total flavonoid content (TFC). HPLC–MS/MS analysis showed that RL was rich in rutin, while CS exhibited a higher quercetin content. Antioxidant activity assessed through ABTS, DPPH, and FRAP assays, was correlated with polyphenolic composition. CS showed the highest antioxidant potential, surpassing green tea by ~39%, as determined via FRAP, while MC and RL displayed capacities comparable to BG. Antibacterial activity assays demonstrated that MC inhibited *Escherichia coli* with a minimum inhibitory concentration (MIC) of 12.5 mg/mL, lower than that of ampicillin, whereas CS was highly active against *Staphylococcus aureus*, with an MIC of 0.39 mg/mL, equivalent to the activity exhibited by tetracycline. Cytotoxicity assays confirmed that the extracts did not reduce human cell viability, supporting the potential of Chilean native shrubs as safe, natural sources of antioxidants and antimicrobials for food and pharmaceutical applications.

## 1. Introduction

In Chile, the use of native plants in traditional medicine is widespread [1]. Nevertheless, scientific evidence supporting their medicinal properties has not been fully revealed and there is a lack of comprehensive chemical characterizations of extracts of these plants [1]. In this context, bioprospecting emerges as a key strategy, as it allows for the scientific validation of the pharmacological and nutraceutical properties of native and endemic plants, while also assessing the safety and/or toxicity [2].

The World Health Organization (WHO) has recognized the relevance of ancestral practices in promoting both individual and collective health, encouraging their progressive integration into modern healthcare systems [3]. In line with this vision, the Chilean Ministry of Health has implemented initiatives to regulate and promote the safe use of medicinal plants [4]. Nevertheless, there remains a pressing need for robust and updated analytical methodologies to scientifically validate the efficacy and safety of these species [1].

The temperate forests of southern Chile are characterized by a high plant biodiversity and are home several to shrub species used in traditional medicine [5,6] such as *Buddleja globosa*, commonly known as matico, which has traditionally been used as an anti-inflammatory and gastroprotective agent [4]. The *Buddleja globosa* exhibits high antioxidant and antibacterial capacity similar to commercial green tea [7]. Other shrub species with folk medicinal uses include *Cissus striata* (voqui rojo), traditionally used to treat wounds and various skin conditions; *Mitraria coccinea* (chilca), applied to bruises, inflammation, and other skin ailments; and *Raukaua laetevirens* (sauco cimarrón), employed as a sudorific and diuretic, and to treat inflammation, burns, and conjunctivitis [4,8,9]. The way these plants are traditionally used suggests that their leaves maybe be a source of bioactive compounds [10,11].

Polyphenolic compounds are known for conferring biological properties like antioxidants and antimicrobial capacities to plant extracts [12,13], but they could also help to reduce the effects of cellular oxidative stress [14,15]. Various studies suggest that the antibacterial capacity of plant extracts against human pathogens such as *E. coli* and *S. aureus* is due to polyphenols [13,16,17]. Plant-based extracts are therefore emerging as alternatives to conventional antibiotics, driven by consumer demand for natural products and the rise in antibiotic-resistant bacteria due to the excessive use of synthetic drugs [18]. However, for extracts to be used in food or pharmaceutical contexts, they must have no adverse effects [12].

In this work, we aimed to evaluate the bioactive properties of three Chilean leaf extracts, focusing on their antibacterial and antioxidant activities, and to assess their effects on human cell viability. These extracts were compared with *Buddleja globosa*, a prominent Chilean medicinal plant, to explore their potential as natural ingredients for use in different stages of agri-food production.

## 2. Materials and Methods

### 2.1. Chemicals and Reagents

The following chemicals were acquired from Sigma–Aldrich (Sigma-Aldrich, St. Louis, MO, USA): aluminum chloride, sodium acetate, sodium carbonate, Folin–Ciocalteu reagent (FC), DPPH (2,2-diphenyl-1-picrylhydrazyl), ABTS (2,2-Azino-bis (3-ethylbenzothiazo-line-6-sulphonic acid)), TPTZ (2,4,6-tripyridyl-s-triazine), FeCl_3_ (ferric chloride hexahydrate), ethanol, quercetin, gallic acid and Trolox (6-hydroxy-2,5,7,8-tetramethylchromane-2-carboxylic acid). DIMEM F12, non-essential amino acids, penicillin-streptomycin, trypsin, and fetal bovine serum were obtained from Biological Industries (Beit HaEmek, Israel). HBSS, PBS, and dichlorofluorescein diacetate, were purchased from Merck (Merck, Darmstadt, Germany).

### 2.2. Sample Collection and Extract Preparation

Leaf samples of *Buddleja globosa* (BG), *Cissus striata* (CS), *Mitraria coccinea* (MC), and *Raukaua laetevirens* (RL) were collected from a site located at 41°00′04.4″ S, 72°34′23.0″ W in Puerto Octay in the Los Lagos region of Chile. Five wild individuals of each species were randomly selected, and fresh, fully developed, leaves were collected. Sampling was conducted during the budburst period, intentionally leaving new buds to ensure subsequent shoot regeneration. In addition, only a small number of leaves per individual was collected so as not to affect their final biomass. These leaves were dried at room temperature, ground using an electric grinder, and passed through a 0.5 mm sieve.

The ground leaves were macerated in 70% ethanol solution at a ratio of 1:10 (weight of plant material to volume of solvent) for 48 h to extract the compounds. The supernatant was recovered, and the procedure was repeated twice. Each extract was rotary evaporated at 38 °C, leaving the extract suspended in water. This was then frozen at −80 °C for 12 h and subsequently freeze-dried (Labconco FreeZone 1 L, Kansas, MO, USA) for 48 h at a condenser temperature of −40 °C and a chamber pressure of 0.06 mbar to obtain a dry product, stored at 4 °C. An aqueous extract containing 100 mg/mL of the plant extract was prepared in water for the subsequent assays.

### 2.3. Quantification of Total Phenolic Content and Total Flavonoid Content

The total phenolic content (TPC) was determined using the Folin–Ciocalteu method with modifications (Singleton et al. [19]). A total of 100 µL of the plant extract solution was mixed with 500 µL of Folin–Ciocalteu reagent (1:10 *v*/*v*) and 400 µL of a 7.5% Na_2_CO_3_ solution for 40 min in the dark at room temperature. Afterwards, 200 μL of the solution was extracted in triplicate, and the absorbance was measured at 760 nm using a FlexA-200 model spectrophotometric plate reader. Quantification was performed by linear regression of a calibration curve constructed using gallic acid (10–150 mg/L). The results were expressed as milligrams of gallic acid equivalent per gram of dry extract and reported as means ± standard deviation (SD).

The total flavonoid content (TFC) was estimated using an aluminum chloride-based methodology. A solution containing 100 μL of the plant extract was mixed with 400 μL of water and 30 μL of 5% NaCO_2_. This solution was left in the dark for five minutes at room temperature before 30 μL of an AlCl_3_ (10%) solution was added. This new solution was left in the dark for 11 min at room temperature and then 200 μL of 1 M NaOH and 240 μL of distilled water were added. A 200 μL aliquot of the solution was then extracted in triplicate for each sample, after which the absorbance was measured at 510 nm using a FlexA-200 spectrophotometric plate reader. The TFC was calculated as mg of catechin equivalents per gram of dry extract using a calibration curve (100–450 mg/L). Values are reported as mean ± standard deviation (SD).

### 2.4. Quantification of Phenolic Compounds Using UHPLC–MS/MS

For the analysis, a chromatographic methodology was implemented using an Eksigent ultra-high-performance liquid chromatography (UHPLC) system coupled to an AB Sciex Triple Quad 4500 tandem mass spectrometer. Separation of the analytes was achieved on a ODS-4 column (10 mm × 4 mm × 3 µm). Chromatographic separation was performed using an aqueous phase consisting of 0.1% formic acid (Mobile Phase A) and an organic phase consisting of methanol (Mobile Phase B). The gradient elution program consisted of the following steps: the initial conditions were set at 85% Mobile Phase A and 15% Mobile Phase B, maintained for 1 min at a flow rate of 0.5 mL/min. The proportion of Mobile Phase B was then linearly increased to 100% at 18 min and held constant until 21 min. The system was subsequently returned to the initial conditions (85% A, 15% B) at 22 min and allowed to equilibrate until 25 min.

Mass spectrometric detection was performed using Analyst 1.6.2 software. The parameters of the chromatographic method are detailed in Table S1. Electrospray ionization (ESI) in negative mode was used for molecular fragmentation. The two major product ions derived from the protonated molecular ion [M+H]^+^ were monitored. The first transition was used for quantification, while the second was used for confirmation of the compound’s identity (Table S1 and Figure S1). Quantification was not performed if either of the transitions was absent. Calibration curves were constructed using commercially available analytical standards of Gallic Acid, Cinnamic Acid, Syringic Acid, Ferulic Acid, Chlorogenic Acid, Sinapic Acid, Caffeic Acid, Cumaric Acid, Catechin, Pinocembrin, Rutin, Quercetin, Quercitrin, Luteolin, Vanillic Acid, Epicatechin, Apigenin, Myricetin.

### 2.5. Antioxidant Capacity

The ABTS assay was performed by mixing 50 μL of each sample with 150 μL of an ABTS solution, the absorbance of which was measured to be 1.1 ± 0.02 at 732 nm. The mixture was then left in darkness at room temperature for 30 min. The absorbance was measured at 732 nm in triplicate for each sample using a FlexA-200 spectrophotometric plate reader. Antioxidant capacity was expressed as milligrams of Trolox equivalents per gram of dry extract using a calibration curve (2–50 mg/L Trolox), with the results expressed as the mean ± standard deviation (SD).

The DPPH radical scavenging assay was conducted by combining 50 μL of each sample with 150 μL of a DPPH solution that had been adjusted to an absorption value of 1.1 ± 0.02 at 517 nm. The mixture was then incubated at ambient temperature for 30 min in the dark. Absorbance was measured at a wavelength of 517 nm, in triplicate, using a FlexA-200 spectrophotometric plate reader. Antioxidant capacity was expressed in milligrams of Trolox equivalents per gram of dry extract, based on a calibration curve (2–60 mg/L Trolox), with results reported as mean ± standard deviation (SD).

The FRAP assay was performed by mixing 50 μL of each sample with 150 μL of FRAP reagent, prepared using 10 mM TPTZ in 40 mM HCl, 20 mM FeCl_3_.6H_2_O, and 300 mM acetate buffer (pH 3.6). The mixture was then incubated at 37 °C for 30 min. Absorbance was measured at a wavelength of 593 nm, with each measurement being recorded in triplicate, sing a FlexA-200 spectrophotometric plate reader. Antioxidant capacity was expressed as milligrams of Trolox equivalents per gram of dry extract, with the help of a calibration curve (2–20 mg/L Trolox), with the results given as mean ± SD.

### 2.6. Antibacterial Activity

Antibacterial activity was determined against *S. aureus* and *E. coli*. Each strain was propagated on Mueller-Hinton agar for 24 h. Colonies from each strain were then dissolved in a saline solution to achieve a viable concentration of 10^8^ CFU/mL, as determined by visual comparison with the McFarland 0.5 standard. The strains were then redistributed into Petri dishes containing Mueller-Hinton agar, and 6 mm holes were cut into the agar to be filled with 100 µL of plant extract solution. The Petri dish cultures were incubated for 24 h at 35 °C before the inhibition diameter was measured. Each extract was evaluated in triplicate.

The minimum inhibitory concentration (MIC) was determined following the method described by EUCAST (2014) [20], with minor adaptations. Briefly, 150 μL of Mueller–Hinton broth and 150 μL of the extract (100 mg/mL) were added to the first column of a 96-well microplate. A two-fold serial dilution was prepared by transferring 150 μL from each well to the next column containing 150 μL of fresh Mueller–Hinton broth, obtaining concentrations reduced by half across each subsequent well. Then, 50 μL of a bacterial suspension (1.5 × 10^6^ CFU/mL) was added to each well. The microplates were incubated at 35 °C for 24 h. After incubation, an aliquot from each well was plated onto Mueller–Hinton agar to confirm bacterial growth. The MIC was defined as the lowest extract concentration at which no visible growth was observed when compared with the control. All assays were performed in triplicate.

### 2.7. Cell Viability Studies by Neutral Red Uptake Assay

Human cancel cell line HCT116 was maintained in DMEM. The medium was supplemented with Fetal Bovine Serum (5% for assays and 10% for growth), 1× non-essential amino acids, 100 U/mL penicillin and 100 μg/mL streptomycin. All cells were cultured in a humidified incubator at 5% CO_2_ atmosphere and 37 °C (NuAire, NU 5700, Plymouth, Devon, UK).

Briefly, HCT116 cells were seeded in a 96-well flat-bottom plate at a density of 1 × 10^4^ cells per well in 100 μL of corresponding media supplemented with 10% FBS. Cells were incubated for 24 h in a humidified incubator at 5% CO_2_ and 37 °C. After incubation, a viability assay was performed according to a previously reported methodology with some modifications [21]. Briefly, media were removed from each plate, and 100 μL of a 2 μg/mL Neutral Red solution in the corresponding medium was added to each well. Cells were incubated for 2 h at 37 °C, gently washed three times with PBS 1X, and then an acidified alcoholic solution was added to extract the incorporated dye. Fluorescence was measured with excitation/emission wavelengths of 530/645 nm, respectively, using a Biotek^®^ Cytation5 reader (Biotek^®^, Winooski, VT, USA).

### 2.8. Kinetic Analysis of Intracellular ROS Generation

Intracellular ROS generation was determined according to the method proposed by Gallardo-Garrido et al., 2020 [21]. Briefly, HCT116 cells were seeded in 96-well flat-bottom fluorescence plates at a density of 5 × 10^4^ cell per well in DMEN with 10% FBD and left overnight. The medium was removed and replaced with fresh, supplemented medium containing 20 μM of DHFDA. After incubation, cells were washed twice with sterile HBSS, and the compounds were added. Fluorescence was measured every 2 min for 1 h with excitation/emission wavelengths of 480/520, respectively, using a Biotek^®^ Cytation5 reader (Biotek^®^, Winooski, VT, USA). The results are expressed as fold changes in fluorescence under each condition over time, normalized to fluorescence of the control over time (slope of condition/slope of control). Positive controls consisted of 20 μM H_2_O_2_.

### 2.9. Statistical Analysis

For each evaluated parameter, a one-way analysis of variance (ANOVA) was performed. Subsequently, Tukey’s test was employed to evaluate significant differences at a confidence level of 95% (*p* < 0.05). All analyses were performed using GraphPad Prism v8 software. PLS models were used to analyze the antioxidant activity and MIC, as well as to determine the VIP scores based on the APCI or MIC data, in relation to the composition of polyphenolic compounds. Compounds with VIP scores greater than 1, determined using the statistics software Statgraphics v8, were considered to make the most significant contributions.

## 3. Results and Discussion

### 3.1. Sustainable Sampling and Species Conservation Considerations

The species used in this study are shrubs with a broad distribution in Chile. Specifically, CS and MC are found from Coquimbo to Magallanes; RL from Maule to Magallanes; and BG from Coquimbo to the Los Lagos Region [8]. According to the Biodiversity Monitoring System of the Chilean Ministry of the Environment [22], none of these species is currently listed under any conservation status in the Los Lagos Region; therefore, the collection of plant material does not pose a risk to native populations.

Although this study focuses on the bioprospecting of native tree species from the temperate rainforest, sustainable sampling criteria were implemented by collecting only a limited portion of leaves per individual and leaving newly formed buds to ensure subsequent shoot regeneration.

In addition, previous studies have reported successful in vitro propagation of BG [23]. The remaining species examined in this work also exhibit favorable traits, such as bud formation during the budburst period, making them potential candidates for in vitro propagation. This approach would allow sustainable production without negatively affecting natural populations.

### 3.2. Compositions of Polyphenolic Compounds

The higher values of total phenolic content (TPC) in the extracts used in this study are due to the use of 70% ethanol as a solvent, which results in a major yield following aqueous extraction [24]. Perfoming the method at lower temperatures also prevent thermal degradation of polyphenolics [7].

**Figure 1:**
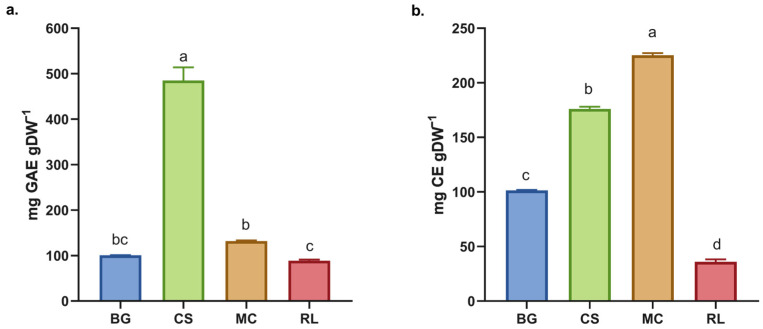
(**a**) Total phenol content (TPC) and (**b**) total flavonoids content (TFC) in the dry extract of native plants. BG = *Buddleja globosa.* CS = *Cissus striata*. MC = *Mitraria coccinea*. RL = *Raukaua laetevirens*. A one-way ANOVA was performed; different letters denote statistically significant differences according to Tukey’s test (*p* < 0.05).

The TPC obtained for RL and MC are similar to those for BG (Figure 1) and contrast with previous findings for BG, for which a yield of 12.2 ± 0.3 mg EAG·g^−1^ dw was reported following boiling water extraction for 20 min [7]. Furthermore, all species exhibited a higher TPC than infusions of commercial green tea under the same experimental conditions [7]. Conversely, CS presented a higher TPC than the other species studied and a higher TPC than extracts obtained using the same methodology, such as *Camellia sinensis* (252.65 ± 4.74 mg EAG·g^−1^), *F. vesca* (148.48 ± 3.12 mg EAG·g^−1^) and *R. fruticosus* (121.87 ± 2.11 mg EAG·g^−1^) [25,26]. These findings indicate that *C. striata* is more effective than tea extracts, which are renowned for their high polyphenol contents [27].

**Figure 2:**
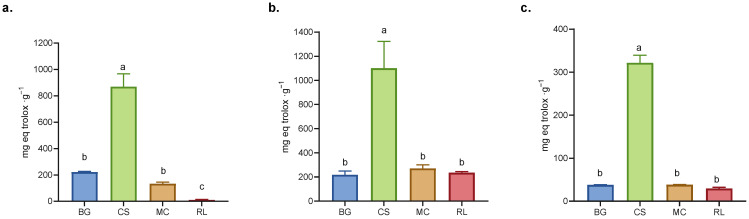
Antioxidant capacity: (**a**) FRAP, (**b**) ABTS, and (**c**) DPPH assays. BG = *Buddleja globosa*. CS = *Cissus striata*. MC = *Mitraria coccinea.* RL = *Raukaua laetevirens*. A one-way ANOVA was performed; different letters denote statistically significant differences according to Tukey’s test (*p* < 0.05).

The total flavonoid content (TFC) varied among the species analyzed, with MC having the highest levels. There were significant differences between all of the species (Figure 2b). When these results are compared with those for the dry extract of *L. litseifolius* [Hance] leaves, it can be seen that its TFC is similar to that of BG, but lower than that of CS and MC [28]. The evaluated species showed higher total flavonoid contents (TFCs) than the dry extracts of *M. lanceolata* [16]. Both *L. litseifolius* and *M. lanceolata* are well known for their medicinal and antioxidant properties, highlighting the functional importance of flavonoids [17,28]. An increased concentration of these compounds may improve the therapeutic efficacy of the extract, given their well-documented antioxidant, anti-inflammatory, and cardioprotective properties [29,30].

In the analysis of polyphenols, performed via HPLC-MS/MS, rutin, quercetin and luteolin stand out as having the highest concentrations in the studied plants (Table 1). The highest concentrations of rutin were observed in RL, while quercetin exhibits the highest concentration in CS. The presence of rutin is particularly relevant due to its established anti-inflammatory activity [30], which supports the use of RL as a traditional natural remedy [4]. In contrast, luteolin levels were higher in MC and BG compared to the other species.

### 3.3. Antioxidant Capacity

The dry extract of CS demonstrated the highest antioxidant capacity in all three assays (ABTS, DPPH, and FRAP), exhibiting significant differences (*p* < 0.05) in comparison to the other three evaluated species (Figure 2). In both the ABTS and DPPH assays, similar response patterns were observed among the extracts, since both assays are based on the detection of antioxidants with proton-donating and electron transfer stabilization mechanisms [31]. However, in the FRAP assay (which specifically measures reducing power), the RL extract showed the lowest activity (Figure 2), suggesting that the presence of electron-donating antioxidants such as caffeic acid, ferulic acid, rutin, luteolin and quercetin contributed less to its overall antioxidant capacity [32,33]. While it could be interpreted that these compounds contribute less because they are present in lower amounts, there is not always a direct relationship between antioxidant capacity and polyphenolic composition, as the compounds may exhibit additive, antagonistic, or synergistic interactions [34].

The dry extract of CS exhibited antioxidant capacity, determined using the FRAP methodology; this activity was approximately 39% higher than that recorded for green tea, a commercial beverage renowned for its antioxidant properties [25]. In the case of BG, previous studies have demonstrated its antioxidant properties, supporting its use in traditional medicine [7,35,36,37]. Conversely, extracts of MC and RL exhibited antioxidant capacities equivalent to those measured for BG (Figure 2). The antioxidant capacities evaluated using DPPH were higher than those observed in arugula leaves for all dry extracts [38]. Concurrently, antioxidant capacity analyses exhibited a consistent pattern in total polyphenol content for each extract, consistent with the extant literature [39,40].

The APCI (Antioxidant Potential Composite Index) integrates and standardizes the results obtained using different methods for evaluating antioxidant capacity, thus allowing for a comprehensive comparison between samples [41]. In the present study, the APCI was higher for CS, while that of MC was comparable to BG but higher than that of RL demonstrated a lower value (see Table S2 for detail). In another study evaluating a variety of polyphenol-rich beverages, the APCI was used to standardize antioxidant capacity across different matrices. In that work, beverages such as pomegranate juice and red wine products already recognized for their strong antioxidant properties exhibited the highest APCI values. This reference is provided only as a conceptual parallel, emphasizing that samples with higher APCI values tend to exhibit greater antioxidant capacity, supports using the APCI as a unifying metric [41].

The Partial Least Squares (PLS) model facilitates the identification of the polyphenols that contribute most significantly to antioxidant assays. It is noteworthy that this contribution is not merely additive but rather is contingent on the type of compound and its proportion in complex mixtures, with the potential for synergistic or antagonistic effects [34]. The PLS model of the APCI identified that the molecules with the greatest influence on antioxidant capacity (VIP-score > 1) were catechin, pinocembrin, quercetin, myricetin, and quercitrin (Figure S2), coinciding with the compounds present in higher concentrations in CS compared to the other extracts (Figure 2). Conversely, compounds such as caffeic acid, luteolin, rutin, and apigenin which exhibited with higher concentrations in the extracts of MC and RL exhibited moderate importance in the APCI (Table 1).

### 3.4. Antibacterial Capacity

**Figure 3:**
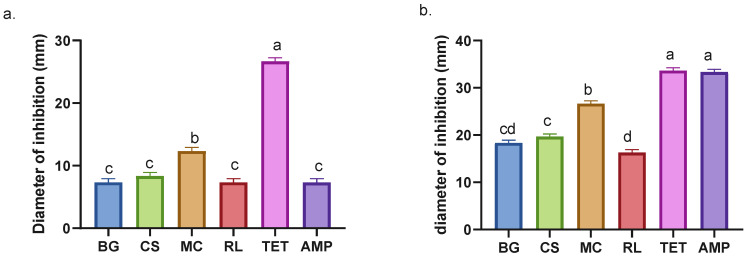
Inhibition halos of plant and antibiotic extracts against *E. coli* (**a**) and *S. aureus* (**b**). BG = *Buddleja globosa.* CS = *Cissus striata*. MC = *Mitraria coccinea*. RL = *Raukaua laetevirens*. TET = Tetracycline. AMP = Ampicillin. A one-way ANOVA was performed; different letters denote statistically significant differences according to Tukey’s test (*p* < 0.05).

Inhibition halos can be used to approximate the effect of the extracts on bacterial growth in vitro. This approximation relies on two factors: the diffusion capacity of the extracts in the medium and the physiological response of the microorganism. The edge of the halo indicates the point at which the minimum inhibitory concentration (MIC) is reached [42]. Evaluation of the inhibition halos against *E. coli* shows that the MC extract exhibits the highest value compared to other species (Figure 3a). Conversely, the other species do not demonstrate significant differences with respect to BG (Figure 3a). Despite its recognized antibacterial properties [36,37], there is no documented history of its utilization in combating *E. coli;* however, other species of the same genus, including *Buddleja indica*, have been observed to impede *E. coli* growth [43].

The patterns observed in the MIC assays are consistent with those described for the inhibition halos (see Table 2 and Figure 3a). For *S. aureus*, CS exhibited a lower MIC value compared to MC, indicating greater antibacterial potency. However, the inhibition assay revealed that MC produced a larger halo than CS. This suggests that the diffusion capacity of the compounds in agar may influence the apparent inhibitory activity rather than reflect their true effectiveness [42].

BG and RL extracts identified as having moderate antibacterial activity against *S. aureus,* while CS and MC were classified as active [44]. Here, it was exhibited that *M. coccinea* obtained the lowest MIC of the four extracts against *E. coli,* achieving a minimum of 12.5 mg/mL, lower than that of ampicillin (Table 1). CS exhibited the lowest MIC value against *S. aureus* (0.39 mg/mL), which was comparable to that of tetracycline and lower than that of ampicillin. The *CS* extract recorded values lower than those reported for *A. indica* and *B. pilosa* [45], as well as the ethanolic extracts of *M. piperita* [46]. It is important to emphasize that this comparison refers exclusively to the antibacterial potential of the extracts and does not constitute an evaluation of the plant species themselves.

PLS models between MIC^−1^ against *E. coli* and polyphenolic compounds show that the compounds with the greatest contribution to this parameter are chlorogenic acid and apigenin, followed by syringic acid, ferulic acid, luteolin, and rutin (Figure S2). In particular, chlorogenic acid has been demonstrated to have bactericidal effects on *E. coli* through direct action on the cell wall and membrane [47], and apigenin has been shown to induce apoptosis-like bacterial death in *E. coli* through the activation of oxidative pathways dependent on the accumulation of reactive oxygen and nitrogen species [48].

Conversely, in the PLS models between MIC^−1^ against *S. aureus*, the compounds that contributed most to this parameter were catechin, pinocembrin, quercetin, myricetin and quercitrin. These compounds exhibited a similar level of influence within the model (Figure S3). This suggests that although they are not the most abundant in the samples (see Table 1), their relative proportions may determine their observed MIC effect against *S. aureus* [49,50]. Possible synergistic interactions within the complex mixtures of the studied extracts should also be considered [51,52].

### 3.5. Cell Viability Studies by Neutral Red Uptake Assay

Assessing cell viability is a fundamental criterion in vitro studies, as it allows for determining whether a drug or a plant extract compromises cell survival [53]. In this study, the assays performed showed that none of the extracts, within the tested concentration range of (1–1000 mg/L, significantly affected the viable cell count of HCT116 cells compared with the control, resulting in the 100% cell viability. This finding is consistent with previous studies that showed that plant-derived extracts and formulations may exhibit low in vitro cytotoxicity, supporting their potential for biomedical, pharmaceutical, or food applications [54]. These results encourage further investigation into the properties of these extracts based on their traditional use.

### 3.6. Intracellular Antioxidant Effect

**Figure 4:**
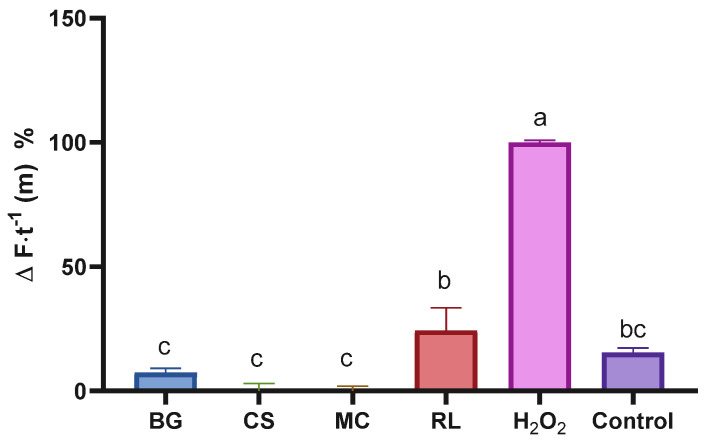
Percentage of intracellular ROS change in cells treated with different plant extracts. Bars with the same letter are not significantly different according to Tukey’s multiple comparison test (*p* < 0.05). Values are expressed relative to the positive control (H_2_O_2_), with ‘Control’ representing untreated cells. BG: *Buddleja globosa*; CS: *Cissus striata*; MC: *Mitraria coccinea*; RL: *Raukaua laetevirens*.

A DHFDA probe was added to the cells with the aim of determining the extracts’ intracellular antioxidant effects. It was observed that the extracts, at the highest concentrations tested, were able to completely suppress (100%) intracellular oxidation induced by H_2_O_2_, demonstrating that their strong antioxidant properties (demonstrated in Figure 2) are also expressed in an intracellular environment (Figure 4). Since ROS are involved in multiple cancer-related pathways, including apoptosis, autophagy, necroptosis, ferroptosis, and oxidative damage to DNA and membranes [55], it is of great importance that potential protective agents against these species act in both extracellular (Figure 2) and intracellular (Figure 4) environments, as observed with our extracts.

## 4. Conclusions

The present study evaluated the chemical composition of four plant species from the rainforest of southern Chile, alongside their antioxidant and antibacterial activity against human pathogens. *Buddleja globosa* (matico) is notable for its antioxidant and antibacterial properties, which support its use in traditional medicines. Moreover, the extraction methodology employed in this study allowed for higher yields and enhanced bioactive properties compared with those used in previous reports.

Species such as *Mitraria coccinea* and *Raukaua laetevirens* exhibited total phenolic compound (TPC) yields and antioxidant capacities comparable to those of matico. In terms of antibacterial activity, *M. coccinea* showed a lower MIC against *E. coli* than ampicillin, whereas Cissus striata exhibited an MIC against *S. aureus* equivalent to that of tetracycline and lower than that of ampicillin.

Importantly, in vitro assays confirmed that the extracts did not induce toxicity in human cells, further supporting their potential application in the pharmaceutical and food industries.

The bioactivities observed in this study demonstrate that *Cissus striata*, *Mitraria coccinea*, and *Raukaua laetevirens* possess strong antioxidant potential and exhibit antibacterial activity against clinically relevant pathogens. These findings provide molecular support for their traditional use and highlight their value as natural sources of compounds with potential applications in human health. While the results of in vitro assays offer preliminary evidence of safety and biological activity, further in vivo studies are needed to evaluate these compounds’ absorption, metabolism, and long-term safety. Such research will be essential for validating their therapeutic potential and advancing the development of natural formulations based on these species.

## Figures and Tables

**Table 1:** Phenolic and flavonoid compound in BG, CS, MC, and RL samples (mg/mL (mean ± SD, *n* = 3)). The letters represent significant differences between samples according to Tukey’s test (*p* < 0.005).

Compound	BG	CS	MC	RL
Syringic acid	0.07 ± 0.02 ^a^	0.05 ± 0.01 ^a^	0.02 ± 0.01 ^a^	0.03 ± 0.01 ^a^
Ferulic acid	0.12 ± 0.02 ^a^	0.10 ± 0.01 ^a^	0.02 ± 0.01 ^a^	0.03 ± 0.01 ^a^
Chlorogenic acid	4.5 ± 0.1 ^b^	ND	10.9 ± 0.1 ^a^	ND
Caffeic acid	1.5 ± 0.1 ^ab^	1.1 ± 0.2 ^c^	1.9 ± 0.2 ^b^	3.2 ± 0.3 ^a^
Catechin	ND	3.73 ± 0.21 ^a^	0.04 ± 0.01 ^b^	0.02 ± 0.01 ^b^
Pinocembrin	0.9 ± 0.1 ^b^	3.7 ± 0.2 ^a^	ND	0.5 ± 0.1 ^b^
Rutin	26.49 ± 0.11 ^b^	0.15 ± 0.02 ^c^	0.53 ± 0.02 ^c^	41.04 ± 0.24 ^a^
Quercetin	8.4 ± 0.3 ^b^	63.0 ± 1.1 ^a^	0.6 ± 0.2 ^d^	3.7 ± 0.4 ^c^
Luteolin	19.5 ± 0.9 ^b^	0.5 ± 0.2 ^d^	24.7 ± 0.6 ^a^	9.4 ± 0.3 ^c^
Apigenin	1.70 ± 0.06 ^b^	0.06 ± 0.01 ^c^	3.10 ± 0.04 ^a^	0.21 ± 0.01 ^c^
Myricetin	ND	0.89 ± 0.03 ^a^	ND	ND
Quercitrin	0.6 ± 0.1 ^d^	14.3 ± 0.5 ^a^	4.8 ± 0.2 ^b^	1.3 ± 0.2 ^c^

**Table 2:** Minimum inhibitory concentration (MIC) of plant extracts and antibiotics against Human pathogenic bacteria. Results are expressed in mg·mL^−1^. Lowercase letters represent significant differences between samples according to Tukey’s test (*p* < 0.005). BG = *Buddleja globosa*. CS = *Cissus striata*. MC = *Mitraria coccinea*. RL = *Raukaua laetevirens*. TET = Tetracycline. AMP = Ampicillin.

Extract or Antibiotic	MIC (mg mL^−1^)	
	*E. coli*	*S. aureus*
BG	50.0 ± 2.4 ^a^	6.25 ± 0.24 ^a^
CS	50.0 ± 2.0 ^a^	0.39 ± 0.01 ^b^
MC	12.5 ± 0.6 ^c^	1.56 ± 0.06 ^b^
RL	50.0 ± 2.2 ^a^	6.25 ± 0.20 ^a^
TET	6.25 ± 0.3 ^d^	0.39 ± 0.02 ^b^
AMP	25.0 ± 0.8 ^b^	1.56 ± 0.05 ^b^

## Data Availability

The original contributions presented in this study are included in the article/Supplementary Materials. Further inquiries can be directed to the corresponding author.

## Supplementary Materials

The following supporting information can be downloaded at: https://www.mdpi.com/article/10.3390/antiox14121488/s1, Table S1: UHPLC–ESI–MS/MS parameters for phenolic compound analysis; Table S2: Antioxidant Power Capacity Index (APCI, expressed as a percentage) of the samples; Figure S1: Representative Mass Spectra transition of analytes in the methodology. (a) chlorogenic acid, (b) apigenin, (c) caffeic acid; Figure S2: VIP scores of the compounds obtained from a PLS model, indicating their contribution to APCI; Figure S3: VIP scores of the compounds obtained from a PLS model, indicating their contribution to 1/MIC against *E. coli*; Figure S4: VIP scores of the compounds obtained from a PLS model, indicating their contribution to 1/MIC against *S. aureus*.
