# Supplementary material for: Southern Chilean Native Plants as Novel Sources of Antioxidant and Antibacterial Extracts
Source: Antioxidants (Basel). 2025 Dec 11;14(12):1488. doi: 10.3390/antiox14121488 (PMC12729486; doi:10.3390/antiox14121488)
Supplement: Supplementary file 1 [file antioxidants-14-01488-s001.zip › antioxidants-3963975-supplementary.pdf]

Supplementary Materials

Southern Chilean Native Plants as Novel Sources of Antioxidant and Antibacterial Extracts

Jesús Hernández [1,†], Yihajara Fuentes [2,†], Eduardo Muñoz-Carvajal [2], Mario Faúndez [3], Miguel Gómez [1], Ady Giordano [2,*] and Gloria Montenegro [1]

[1] Laboratorio de Productos Naturales, Departamento de Ciencias Vegetales, Facultad de Agronomía y Sistemas Naturales, Pontificia Universidad Católica de Chile, Santiago 782-0436, Chile
[2] Departamento de Química Inorgánica, Escuela de Química, Facultad de Química y de Farmacia, Pontificia Universidad Católica de Chile, Santiago 782-0436, Chile
[3] Departamento de Farmacia, Escuela de Química y Farmacia, Facultad de Química y de Farmacia, Pontificia Universidad Católica de Chile, Santiago 782-0436, Chile
* Correspondence: Correspondence: agiordano@uc.cl
† These authors contributed equally to this work.

Methodology

Table S1. UHPLC–ESI–MS/MS Parameters for Phenolic Compound Analysis.

Analyte	Mass Q1	Mass Q3	DP	CE	CXP
**Gallic Acid**	168	124	-70,0	-70,0	-7,0
	168	78	-70,0	-70,0	-15,0
**Cinnamic Acid**	146	103	-55,0	-55,0	-5,0
	146	76	-55,0	-55,0	-7,0
**Syringic Acid**	196	181	-65,0	-65,0	-5,0
	196	122	-65,0	-65,0	-7,0
**Ferulic Acid**	192	146	-5,0	-5,0	-5,0
	192	102	-5,0	-5,0	-11,0
**Chlorogenic Acid**	353	191	-75,0	-75,0	-5,0
	353	85	-75,0	-75,0	-9,0
**Sinapic Acid**	223	207	-75,0	-75,0	-7,0
	223	148	-75,0	-75,0	-13,0
**Caffeic Acid**	178	135	-70,0	-70,0	-5,0
	178	133	-70,0	-70,0	-7,0
**Cumaric Acid**	162	119	-70,0	-70,0	-5,0
	162	92	-70,0	-70,0	-25,0
**Catechin**	289	245	-100,0	-100,0	-13,0
	289	108	-100,0	-100,0	-7,0
**Pinocembrin**	255	212	-95,0	-95,0	-7,0
	255	151	-95,0	-95,0	-7,0
**Rutin**	609	299	-170,0	-170,0	-13,0
	609	300	-170,0	-170,0	-9,0
**Quercetin**	301	150	-115,0	-115,0	-13,0
	301	178	-115,0	-115,0	-11,0
**Quercitrin**	447	299	-85,0	-85,0	-11,0
	447	178	-85,0	-85,0	-7,0
**Luteolin**	285	133	-125,0	-125,0	-5,0
	285	150	-125,0	-125,0	-11,0
**Vanillic Acid**	166	122	-50,0	-50,0	-9,0
	166	151	-50,0	-50,0	-5,0
**Epicatechin**	288	244	-110,0	-110,0	-19,0
	288	109	-110,0	-110,0	-7,0
**Apigenin**	268	117	-130,0	-130,0	-9,0
	268	150	-130,0	-130,0	-5,0
**Myricetin**	316	150	-120,0	-120,0	-13,0
	316	178	-120,0	-120,0	-11,0

Q1 = precursor ion mass; Q3 = product ion mass; DP = declustering potential; CE = collision energy; CXP = collision cell exit potential. All parameters were optimized using electrospray ionization (ESI) in negative mode under multiple reaction monitoring (MRM) conditions.

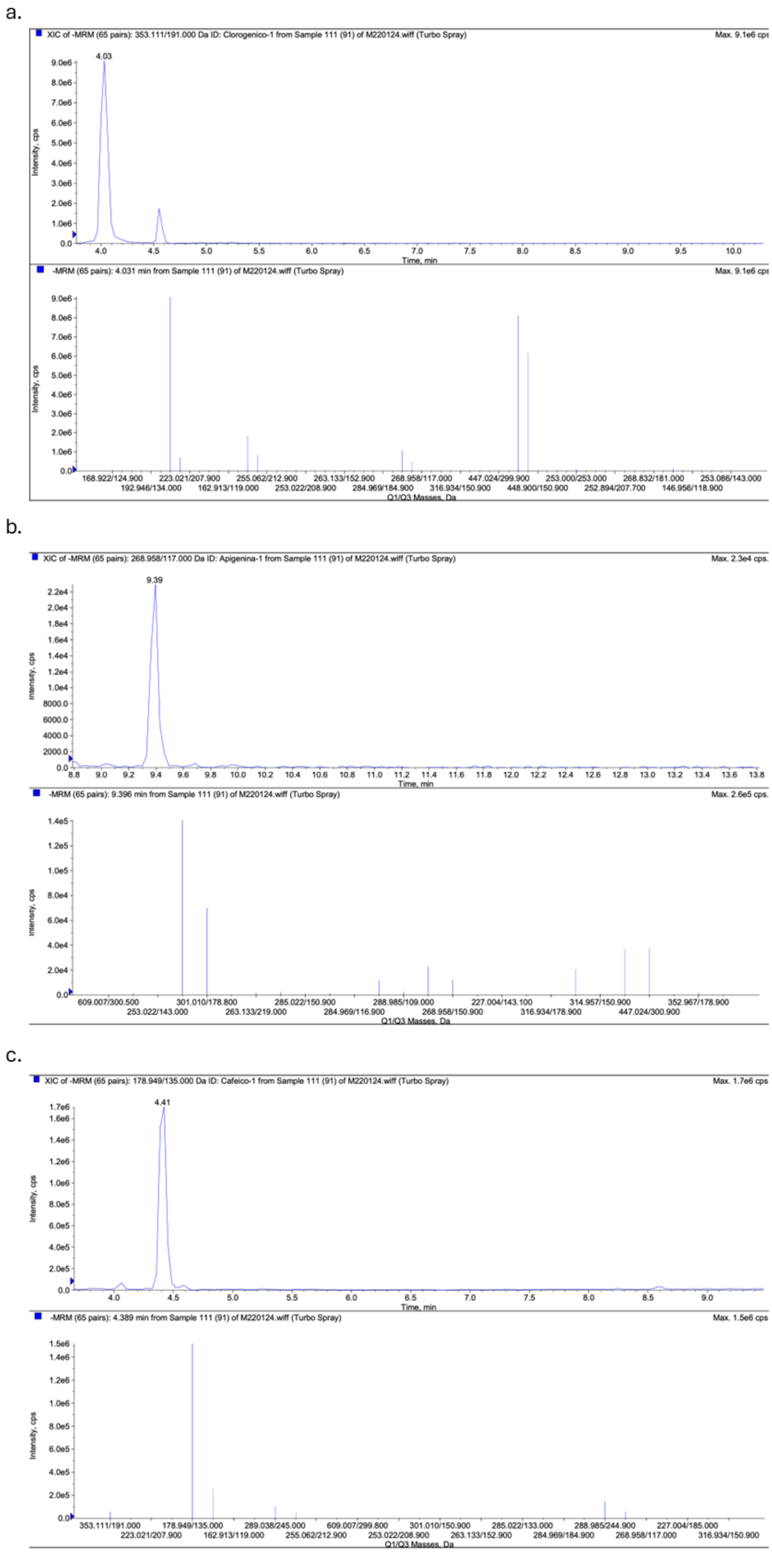

Figure S1. Representative Mass Spectra transition of analytes in the methodology. (**a**) chlorogenic acid, (**b**) apigenin, (**c**) caffeic acid.

Results

Table S2. Antioxidant Power Capacity Index (APCI, expressed as a percentage) of the samples. BG = *Buddleja globosa*; CS = *Cissus striata*; MC = *Mitraria coccinea*; RL = *Raukaua laetevirens*.

Species	APCI
BG	19.01
CS	100.00
MC	17.31
RL	10.59

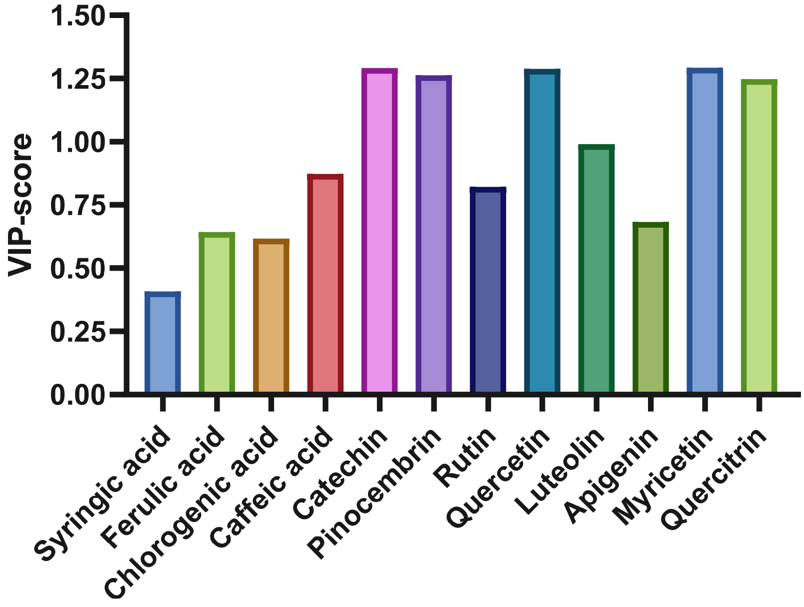

Figure S2. VIP scores of the compounds obtained from a PLS model, indicating their contribution to APCI.

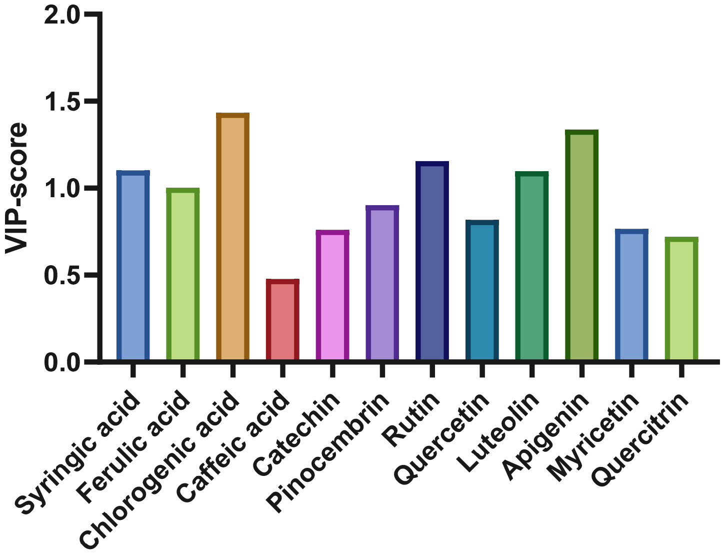

Figure S3. VIP scores of the compounds obtained from a PLS model, indicating their contribution to 1/MIC against *E. coli*.

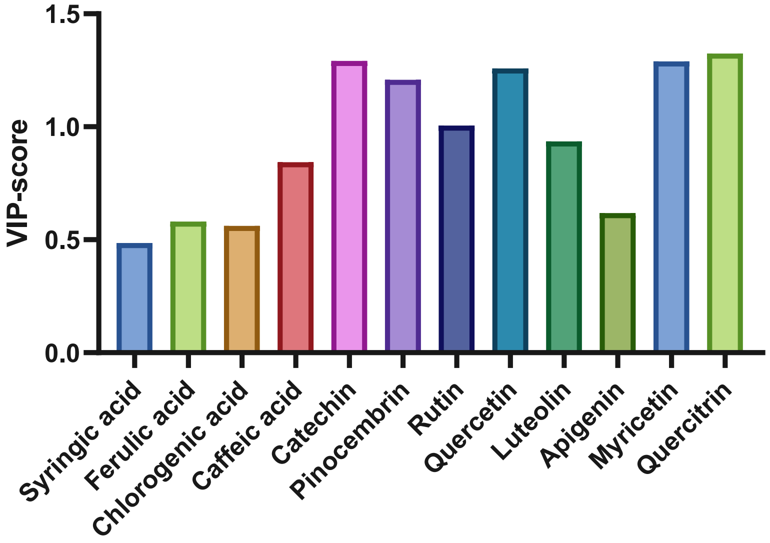

Figure S4. VIP scores of the compounds obtained from a PLS model, indicating their contribution to 1/MIC against *S. aureus*.
